# Free Fatty Acids and Their Metabolism Affect Function and Survival of Podocytes

**DOI:** 10.3389/fendo.2014.00186

**Published:** 2014-10-27

**Authors:** Jonas Sieber, Andreas Werner Jehle

**Affiliations:** ^1^Division of Nephrology, Massachusetts General Hospital, Harvard Medical School, Boston, MA, USA; ^2^Molecular Nephrology, Department of Biomedicine, University Hospital Basel, Basel, Switzerland; ^3^Transplantation Immunology and Nephrology, Department of Internal Medicine, University Hospital Basel, Basel, Switzerland

**Keywords:** podocyte, diabetic nephropathy, saturated and monounsaturated free fatty acids, lipid metabolism, lipotoxicity, endoplasmic reticulum stress, β-oxidation

## Abstract

Podocyte injury and loss critically contribute to the pathogenesis of proteinuric kidney diseases including diabetic nephropathy. Deregulated lipid metabolism with disturbed free fatty acid (FFA) metabolism is a characteristic of metabolically unhealthy obesity and type 2 diabetes and likely contributes to end-stage kidney disease irrespective of the underlying kidney disease. In the current review, we summarize recent findings related to FFAs and altered renal FFA metabolism with a special focus on podocytes. We will outline the opposing effects of saturated and monounsaturated FFAs and a particular emphasis will be given to the underlying molecular mechanisms involving insulin resistance and endoplasmic reticulum homeostasis. Finally, recent data suggesting a critical role of renal FFA metabolism to adapt to an altered lipid environment will be discussed.

## Introduction

Diabetic nephropathy (DN) is the major cause of end-stage renal disease, and most affected patients have type 2 diabetes (1, 2). Injury and loss of the glomerular epithelial cells or podocytes are critical in the pathogenesis of DN (3–6). Importantly, the epidemic of DN and type 2 diabetes parallels the obesity epidemic (7, 8), which also drives other causes of chronic kidney disease (CKD) including obesity-related glomerulopathy and secondary focal segmental glomerulosclerosis [reviewed in Ref. (9)]. Obesity-related glomerulopathy has been found to be more associated with serum triglyceride (TG) levels and ectopic lipid accumulation in the kidney than with obesity *per se* (10). Lipid excess and deregulated lipid metabolism in the kidney are increasingly recognized as pathogenic factors not only in the development and progression of obesity-related renal disease and DN but they may also contribute to CKD irrespective of the underlying pathology (9). Excessive lipid droplets can be found in different renal cell types including podocytes (9, 11). Accumulation of lipids in non-adipose tissues can contribute to cellular dysfunction and cell death, a phenomenon that is called lipotoxicity. Elevated plasma free fatty acids (FFAs) and disturbed FFA metabolism critically contribute to lipotoxicity. However, to which extent and how the various FFAs and their metabolites such as diacylglycerols (DAGs) and TGs are pathogenic or are even part of a protective, adaptive process is under debate. Recent data also indicate that cholesterol accumulation in podocytes plays a critical pathogenic role in DN and contributes to lipotoxicity (12).

This review will highlight the potential consequences of altered FFA levels and disturbed FFA metabolism with a special focus on podocytes. If adaptation fails, lipotoxicity with insulin resistance and endoplasmic reticulum (ER) stress may ultimately result in podocyte death. Recent advances in the underlying cellular processes will be summarized and may help to foster further research to find and translate novel therapeutic strategies. In addition, a particular emphasis will be laid on discussing cellular adaptive responses, which might be interesting targets in supporting podocytes dealing with an altered lipid environment.

## Plasma Free Fatty Acids

A main source of lipids is plasma FFAs, which are hydrolyzed and relieved from adipocyte TG stores and carried by plasma albumin to provide energy for tissues during fasting (13). In addition, fatty acids hydrolyzed from liver-derived low density lipoprotein TG by lipoprotein lipases are also contributing to the FFA pool in tissues (14). FFA levels are underlying diurnal fluctuations with low postprandial levels where most FFAs are taken up by the adipose tissue and increased levels during states of fasting (15). In contrast to the general belief, obesity is not generally associated with increased fasting FFA levels, and this association only exists in certain groups of obese and type 2 diabetic patients (16). Elevated FFA levels are associated with and may result from insulin resistance and increased lipolysis (15, 17–19). *Vice versa*, insulin resistance can be the result of elevated FFAs (20). In addition, adipocytes of obese individuals can become defective in FFA uptake, which contributes to elevated FFA levels and promotes ectopic fat deposition (21). This might be of interest as plasma FFA composition partially reflects dietary fatty acid composition (22) and saturated FFAs (SFAs), and monounsaturated FFAs (MUFAs) have distinct effects on cell metabolism and function. In this context, a recent intervention trial is of interest, which demonstrated that a Mediterranean diet enriched with extra-virgin olive oil is effective in the primary prevention of cardiovascular diseases and diabetes (23, 24), and it has been suggested that this beneficial effect at least in part results from the high content of MUFAs in olive oil (24). Further interventional studies are warranted to test whether dietary shifting of the FFA balance toward unsaturated FFAs can prevent and delay the progression of obesity-related renal diseases and DN.

## Opposing Effects of SFAs and MUFAs

**Figure 1:**
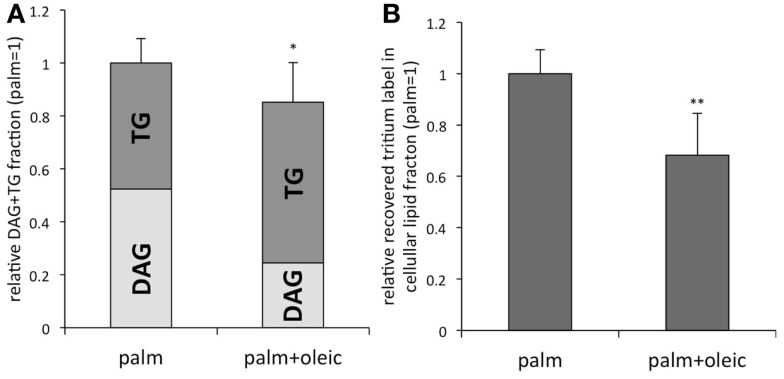
**(A)** Tracing studies with tritium-labeled palmitic acid reveal that MUFAs such as oleic acid slightly reduce the total amount of [^3^H]palmitic acid containing DAG + TG in the cellular lipid fraction. In addition, oleic acid leads to a preferential incorporation of [^3^H]palmitic acid into TG vs. DAG. **(B)** Total amount of tritium-labeled metabolites recovered in the total cellular lipid fraction was decreased in the presence of oleic acid. Adapted from Sieber et al. (37). *n* = 9; *p < 0.01, **p < 0.00001.

The SFAs, palmitic and stearic acid, together with the MUFA oleic acid account for 70–80% of plasma FFAs (25, 26). Interestingly, in most cell types including podocytes mainly SFAs are inducing lipotoxicity such as insulin resistance and cell death (27, 28). By contrast, MUFAs can prevent SFA induced lipotoxicity (Table 1), i.e., an equimolar combination of palmitic and oleic acid does not lead to podocytes death (28). Most of the current understanding of the opposing effects has been derived from studies with hepatocytes, muscle cells, and pancreatic β-cells linking the detrimental actions of SFAs to SFA-derived metabolites such as DAGs and ceramide (29). DAG-mediated activation of protein kinase C (PKC) δ and increased levels of ceramide are associated with the intrinsic mitochondrial apoptotic pathway, e.g., increased mitochondrial membrane permeability and cytochrome *c* release (30–32). Cytochrome *c* release is also observed in palmitic acid treated podocytes (preliminary data). Of note, some studies show partially conflicting findings in the light of the effects of increased ceramide synthesis, TG accumulation, and β-oxidation with its associated reactive oxygen species (ROS) (33–36). In human podocytes, ceramide accumulation has been linked to palmitic acid-induced insulin resistance (27); however, the ceramide synthase inhibitor fumonisin B1 is not ameliorating survival of murine podocytes exposed to palmitic acid (37). Of note, tracing studies with tritium-labeled palmitic acid could show that MUFAs, such as oleic acid, slightly but significantly reduce the total amount of intracellular [^3^H]palmitic acid containing DAG and TG (Figure 1A). More importantly, oleic acid leads to preferential incorporation of [^3^H]palmitic acid derived metabolites into TGs, which is accompanied by a reduction of the [H^3^]palmitic acid containing DAG fraction [Figure 1A, adapted from Sieber et al. (37)]. Furthermore, oleic acid stimulates β-oxidation of palmitic acid (37) and may be beneficial simply by reducing the levels of palmitic acid and its toxic metabolites (38), which might be reflected by the overall decreased recovery of tritium derived from labeled palmitic acid in the total cellular lipid fraction (Figure 1B). In summary, although the beneficial effects of MUFAs are not completely understood, recent work including studies in podocytes points toward facilitated incorporation of palmitic and its metabolites into TGs and increased palmitic acid β-oxidation, which have been postulated to prevent from accumulation of toxic SFA metabolites (29, 35, 37).

**Table 1:** **Beneficial effects of MUFAs on podocytes treated with palmitic acid**.

	Action of MUFAs (e.g., oleic acid) in podocytes treated with palmitic acid	Reference
Cell viability	Prevention of podocyte death	(28)
ER stress/ UPR	Induction of the adaptive UPR (e.g., BiP)	(28)
	Prevention of CHOP induction	(28)
Insulin resistance	Improved insulin sensitivity	Unpublished observation
Lipid metabolism	Increased fatty acid β-oxidation	(37 )
	Reduced accumulation of palmitic acid in DAG	Figure 1A (37)
	Preferential incorporation of palmitic acid into TG	Figure 1A (37)
	Increased DGAT gene expression	Unpublished observation
	Reduced accumulation of palmitic acid derived metabolites in the total lipid fraction	Figure 1B (37)

### Insulin resistance

In human (27) and murine (preliminary data) podocytes, palmitic acid induces insulin resistance. Studies in hepatocytes and skeletal muscle cells linked palmitic acid-induced insulin resistance to alternate serine/threonine phosphorylation of insulin receptor substrate (IRS) 1, 2, and AKT by either ROS-mediated JNK (c-Jun NH_2_-terminal kinase) activation or by DAG activation of PKC (39–41). In podocytes, insulin signaling could be ameliorated by JNK inhibition (preliminary data). However, this was not sufficient to ameliorate survival of podocytes chronically exposed to palmitic acid (preliminary data). Of note, JNK activation is also downstream of disturbed ER homeostasis referred to as ER stress, which has been causatively linked to palmitic acid-induced podocyte death (28) (see below). Reduced insulin sensitivity is observed in glomeruli of obese and diabetic rats (42) and normal insulin signaling seems critical for podocyte function and survival as podocyte-specific insulin receptor knockout mice develop albuminuria and glomerulosclerosis (43). In summary, these findings point toward a potential FFA-mediated role of insulin resistance in the development and progression of obesity-related renal disease and DN.

### ER stress

FFA mediated ER stress has been associated with the pathogenesis of obesity and type 2 diabetes where it is extensively studied in the light of pancreatic β-cell failure and the onset of type 2 diabetes (44, 45). ER stress is also observed in the tubulointerstitial and glomerular compartment of renal biopsies obtained from patients with DN (28, 46, 47). Importantly, ameliorating ER stress has been shown to attenuate DN in a type 1 diabetes mouse model (48, 49).

Disturbed ER homeostasis decreases the ER folding capacity and thereby leads to accumulation of unfolded and misfolded proteins, which in turn initiates the unfolded protein response (UPR). The UPR is primarily an adaptive response to maintain proper ER function (50, 51) and involves three signaling branches that are mediated by the ER transmembrane receptors PERK (PKR-like ER kinase), ATF6 (activating transcription factor 6), and IRE-1 (inositol-requiring enzyme 1), which ultimately lead to translational attenuation, ER-associated protein degradation, increased ER chaperone expression, and ER membrane synthesis. If ER stress persists, cells initiate apoptosis, which has been linked to the proapoptotic transcription factor C/EBP homologous protein (CHOP) (52–55). In podocytes, palmitic acid-induced ER stress results in the induction of several UPR markers/effectors, including the ER chaperone heavy chain binding protein (BiP), Gadd34 (Growth arrest and DNA damage-inducible protein), as well as alternate splicing of X-box binding protein 1 (Xbp1), and upregulation of CHOP [(28) and unpublished results]. Contrariwise, the monounsaturated palmitoleic and oleic acids alone upregulated BiP but not CHOP (28). As BiP is known to protect from palmitic acid-induced apoptosis (56), the upregulation of the ER chaperone BiP by MUFAs likely contributes to their beneficial effect. Furthermore, MUFAs attenuate palmitic acid-induced upregulation of CHOP in podocytes, and gene silencing of CHOP protects against palmitic acid-induced podocyte death, which points to a causative role for CHOP (28). A recent study suggests that the detrimental effects of SFAs are linked to activation of mTORC1 (mammalian target of rapamycin complex 1) and subsequent CHOP upregulation (57). Similarly, CHOP deficient mice are protected from DN as well as age-related albuminuria (58). Surprisingly, however, CHOP levels were either unchanged or significantly downregulated in the tubulointerstitial (46) and glomerular (28) compartment of renal biopsies obtained from patients with DN, which could indicate that CHOP positive cells may die and be removed from the tissue. An alternative explanation might be that during the progression of DN, there is a selection of podocytes adapted to the altered environment. The apoptotic actions of CHOP are not completely understood (59); however, proapoptotic targets include GADD34 (60), DR5 (TRAIL Receptor-2) (61), and ERO1α (ER oxidoreductase-1α) (62). In addition, CHOP has been associated with downregulation of anti-apoptotic Bcl-2 (63).

Saturated FFA-mediated ER stress and subsequent UPR have been associated with altered ER membrane composition and disrupted ER integrity (64). In addition of being activated by unfolded proteins, the ER stress sensor IRE-1 has been shown to be sensitive to alterations in ER membrane lipid composition (65). This is as in β-cells, palmitic acid-induced ER stress does not correlate with unfolded proteins (66). Interestingly, in preliminary experiments, specific IRE-1 inhibition with the small molecule compound 4μ8C attenuates palmitic acid-induced podocyte death, which is in accordance with the crucial role of IRE-1 in determining cell fate (67, 68).

## Cellular Adaptive Responses: Regulation of β-Oxidation

SFA lipotoxicity has been linked to lipid accumulation including TGs (69, 70) and increased ROS derived from enhanced β-oxidation (39). However, recent findings indicate that increased FFA β-oxidation as well as TG synthesis may not have to be harmful in any case, but may reflect a protective adaptive response helping podocytes to deal with elevated FFA levels.

Specifically, increasing fatty acid oxidation reduces the susceptibility of podocytes to palmitic acid (38). Fatty acid β-oxidation can be enhanced by AICAR (5-Aminoimidazole-4-carboxamide ribonucleotide), an agonist of the energy-sensor AMPK (AMP-activated protein kinase), which inactivates the acetyl-CoA carboxylase (ACC) and thereby reduces levels of the natural carnitine palmitoyltransferase 1 (CPT1) inhibitor malonyl-CoA. The protective effect of Aicar on palmitic acid could be reversed by inhibiting CPT1, the rate-limiting enzyme of fatty acid β-oxidation. Similarly, ACC-silenced podocytes were less susceptible to palmitic acid (38). Importantly, several recent genome-wide association studies in type 2 diabetic patients found a single-nucleotide polymorphism in a non-coding region of ACC2 to be strongly associated with proteinuria (71–73). The polymorphism is associated with increased ACC2 expression (71), which tends to increased levels of the CPT1 inhibitor malonyl-CoA and diminished fatty acid β-oxidation capacity.

Of interest, the adipocyte-derived hormone adiponectin, a physiological activator of AMPK signaling, slightly improves survival of podocytes treated with palmitic acid (38). However, the protective effect could only be seen in the presence of high glucose, which is known to reduce AMPK signaling (74) and thereby allowed to uncover the effect of adiponectin (38). This observation is in line with and gives a further explanation for the known renoprotective effect of adiponectin (74).

A recent study found an altered gene expression profile of key enzymes of fatty acid metabolism in glomeruli of patients with DN (37). Of note, an upregulation of all three CPT1 isoforms and a downregulation of ACC2 were found (37), which both suggest disposition for increased fatty oxidation. An increase in fatty acid oxidation likely contributes to a protective, adaptive response by decreasing the load of toxic SFAs (38). A second study, however, found a decreased expression of CPT1 in DN (11). However, these results were obtained from whole kidneys (11) and not from glomerular extracts (37), which may explain these discrepancies.

## Cellular Adaptive Responses: SCD1 Expression and Regulation of TG Synthesis

Both aforementioned gene expression analyses found an increased expression of stearoyl-CoA desaturases (SCD) 1 in diabetic kidneys (11, 37) and by immunohistochemistry the glomerular upregulation of SCD1 could be mainly localized to podocytes (37). SCDs desaturate SFAs to MUFAs and thereby provide acyl-CoA:diacylglycerolacyltransferases (DGATs), which catalyze the final step in TG synthesis, with their preferred substrates (75, 76). In glomeruli, the expression of DGAT1 was also found to be increased (37), and together these results indicate not only facilitated conversion of toxic SFAs to MUFAs but also stimulation of TG synthesis.

**Figure 2:**
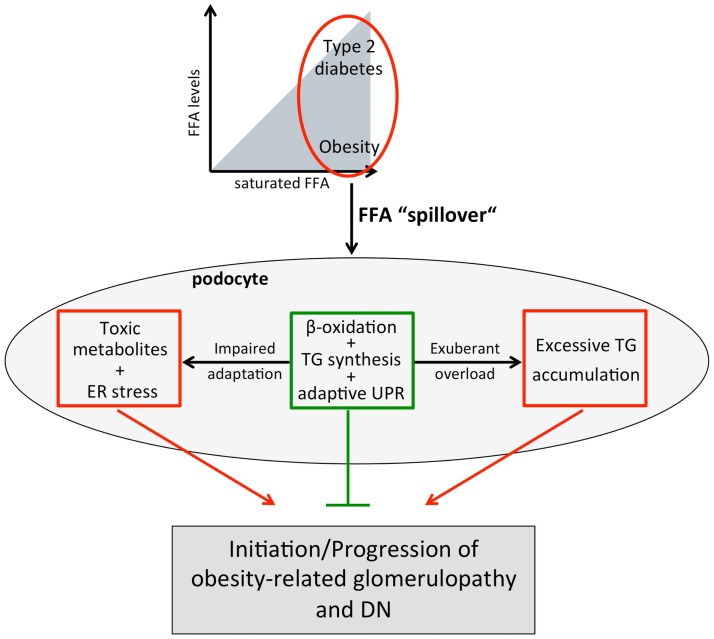
**Working model for increased plasma FFA levels and/or a shift toward SFAs on podocytes**. In obesity and type 2 diabetes, increased adipose tissue lipolysis and/or a FFA uptake defect of adipocytes together with increased dietary FFA intake results in elevated plasma FFAs and a “spillover” of FFAs to non-adipose tissues including the kidney and podocytes. Podocytes may adapt to the altered lipid environment by upregulating fatty acid β-oxidation, TG synthesis, and the adaptive branch of the UPR. However, impaired adaptive capacity (e.g., genetic) or chronic “overload” leading to accumulation of toxic FFA metabolites and/or excessive TG storage may lead to diminished podocyte function and ultimately podocyte death resulting in obesity-related glomerulopathy and DN.

In obesity, accumulation of TG has been linked to peripheral lipotoxicity (69, 70); however, TG storage might not be pathogenic *per se* as endurance-trained athletes show higher TG levels as well as higher insulin sensitivity in skeletal muscle, a phenomenon known as the “athletes’ paradox” (77, 78). Also, transgenic mice overexpressing the TG-synthesizing enzyme DGAT1 in the heart have an increased TG content, but improved cardiac function (79). Moreover, mice overexpressing chREBP (carbohydrate response element-binding protein) fed a high-fat diet have increased hepatic levels of SCD1 and DGAT1 and show elevated insulin sensitivity despite increased hepatic steatosis (80). On the other hand, at some point, renal lipid accumulation may become harmful and a reduction in lipid overload by farnesoid X receptor agonists, which reduce the lipid synthesis regulator SREBP-1 (sterol regulatory element-binding protein), has been shown to slow the progression of DN (81) and obesity-induced nephropathy (82). It can be hypothesized that the extent, location, and context of TG deposition determine lipotoxicity, and specifically, if it prevents accumulation of even more toxic SFA metabolites such as ceramide and DAGs, the beneficial effects may outweigh [Figure 2 (35)].

Pharmacological stimulation of SCDs by liver X receptor agonists protects podocytes from palmitic acid-induced cell death and this protective effect is lost in podocytes deficient of SCD1 and 2. Also, SCD1 and 2 double deficient podocytes are more susceptible to palmitic acid, but genetic overexpression of SCD1 is protective. These data suggest that the abovementioned increased expression of SCD1 in podocytes likely is part of a protective, adaptive response, which helps podocytes dealing with FFAs.

## Summary and Conclusion

Disordered lipid metabolism and renal lipid accumulation are not only associated with obesity-related renal disease and DN but there is also growing insight that they contribute to the disease process. Recent human and experimental studies suggest that disturbed FFA metabolism plays a critical role in disordered lipid metabolism. As podocytes are highly susceptible to the saturated palmitic acid, but protected by MUFAs as well as increased expression of SCDs, the upregulation of SCD1 in podocytes of diabetic kidneys likely is part of a protective mechanism against SFAs and their toxic metabolites. The toxicity of SFAs in podocytes is partially explained by induction of ER stress and insulin resistance. Several mechanisms can explain the protective effect of MUFAs in podocytes including attenuation of the palmitic acid-induced CHOP upregulation and stimulation of fatty acid β-oxidation. The potentially crucial importance of β-oxidation is supported by genome-wide association studies in type 2 diabetic patients, which found that a single-nucleotide polymorphism in ACC2 favoring impairment of β-oxidation is associated with proteinuria. The observation that the protective effect of MUFAs is associated with a shift of palmitic acid from DAG to TG suggests that “limited” accumulation of TGs in podocytes does not have to be deleterious, but may prevent accumulation of even more toxic FFA metabolites. In conclusion, recent data not only suggest that podocytes are highly susceptible to FFAs, but they have also the potential to adapt to a certain extent to an altered lipid environment (Figure 2). In light of these findings, obese or type 2 diabetic patients with reduced ability for an adaptive response to a disturbed lipid metabolism are likely more prone to develop proteinuria and CKD (Figure 2). Therefore, novel strategies supporting podocytes in their adaptive responses may help to prevent and delay progression of CKD.

## Conflict of Interest Statement

The authors declare that the research was conducted in the absence of any commercial or financial relationships that could be construed as a potential conflict of interest.

## Abbreviations

ACC, acetyl-CoA carboxylase; AMPK, AMP-activated protein kinase; ATF6, activating transcription factor 6; Bcl-2, B-cell lymphoma 2; BiP, heavy chain binding protein; CHOP, C/EBP homologous protein; chREBP, carbohydrate responsive element-binding protein; CKD, chronic kidney disease; CPT, carnitine palmitoyltransferase; DAG, diacylglycerol; DGAT, acyl-CoA:diacylglycerolacyltransferase; DN, diabetic nephropathy; DR5, death receptor 5; ER, endoplasmic reticulum; ERO1, ER oxidoreductase 1; FFA, free fatty acid; Gadd34, growth arrest and DNA damage-inducible protein 34; IRE1, inositol-requiring enzyme 1; IRS, insulin receptor substrate; JNK, c-Jun NH_2_-terminal kinase; mTORC1, mammalian target of rapamycin complex 1; MUFA, monounsaturated fatty acid; PERK, PKR(protein kinase RNA)-like ER kinase; PKC, protein kinase C; ROS, reactive oxygen species; SCD, stearoyl-CoA desaturase; SFA, saturated fatty acid; SREBP, sterol regulatory element-binding protein; TG, triglyceride; TRAIL, tumor necrosis factor related apoptosis inducing ligand; UPR, unfolded protein response; Xbp1, X-box binding protein.

## References

[1] USRDS: the United States renal data system. Am J Kidney Dis (2003) 42(6 Suppl 5):1–230 14655174

[2] Locatelli F Pozzoni P Del Vecchio L. Renal replacement therapy in patients with diabetes and end-stage renal disease. J Am Soc Nephrol (2004) 15(Suppl 1):S25–9. 10.1097/01.ASN.0000093239.32602.04 14684667

[3] Dalla Vestra M Masiero A Roiter AM Saller A Crepaldi G Fioretto P. Is podocyte injury relevant in diabetic nephropathy? Studies in patients with type 2 diabetes. Diabetes (2003) 52(4):1031–5. 10.2337/diabetes.52.4.1031 12663476

[4] Pagtalunan ME Miller PL Jumping-Eagle S Nelson RG Myers BD Rennke HG Podocyte loss and progressive glomerular injury in type II diabetes. J Clin Invest (1997) 99(2):342–8. 10.1172/JCI119163 9006003 PMC507802

[5] White KE Bilous RW Diabiopsies Study Group. Structural alterations to the podocyte are related to proteinuria in type 2 diabetic patients. Nephrol Dial Transplant (2004) 19(6):1437–40. 10.1093/ndt/gfh129 14993494

[6] Chen HM Liu ZH Zeng CH Li SJ Wang QW Li LS. Podocyte lesions in patients with obesity-related glomerulopathy. Am J Kidney Dis (2006) 48(5):772–9. 10.1053/j.ajkd.2006.04.079 17059996

[7] Coresh J Selvin E Stevens LA Manzi J Kusek JW Eggers P Prevalence of chronic kidney disease in the United States. JAMA (2007) 298(17):2038–47. 10.1001/jama.298.17.2038 17986697

[8] Eckardt KU Coresh J Devuyst O Johnson RJ Köttgen A Levey AS Evolving importance of kidney disease: from subspecialty to global health burden. Lancet (2013) 382(9887):158–69. 10.1016/S0140-6736(13)60439-0 23727165

[9] de Vries AP Ruggenenti P Ruan XZ Praga M Cruzado JM Bajema IM Fatty kidney: emerging role of ectopic lipid in obesity-related renal disease. Lancet Diabetes Endocrinol (2014) 2(5):417–26. 10.1016/S2213-8587(14)70065-8 24795255

[10] Verani RR. Obesity-associated focal segmental glomerulosclerosis: pathological features of the lesion and relationship with cardiomegaly and hyperlipidemia. Am J Kidney Dis (1992) 20(6):629–34. 10.1016/S0272-6386(12)70230-5 1462993

[11] Herman-Edelstein M Scherzer P Tobar A Levi M Gafter U. Altered renal lipid metabolism and renal lipid accumulation in human diabetic nephropathy. J Lipid Res (2014) 55(3):561–72. 10.1194/jlr.P040501 24371263 PMC3934740

[12] Merscher-Gomez S Guzman J Pedigo CE Lehto M Aguillon-Prada R Mendez A Cyclodextrin protects podocytes in diabetic kidney disease. Diabetes (2013) 62(11):3817–27. 10.2337/db13-0399 23835338 PMC3806621

[13] Lafontan M Langin D. Lipolysis and lipid mobilization in human adipose tissue. Prog Lipid Res (2009) 48(5):275–97. 10.1016/j.plipres.2009.05.001 19464318

[14] Teusink B Voshol PJ Dahlmans VE Rensen PC Pijl H Romijn JA Contribution of fatty acids released from lipolysis of plasma triglycerides to total plasma fatty acid flux and tissue-specific fatty acid uptake. Diabetes (2003) 52(3):614–20. 10.2337/diabetes.52.3.614 12606500

[15] Reaven GM Hollenbeck C Jeng CY Wu MS Chen YD. Measurement of plasma glucose, free fatty acid, lactate, and insulin for 24 h in patients with NIDDM. Diabetes (1988) 37(8):1020–4. 10.2337/diabetes.37.1.28 3292322

[16] Karpe F Dickmann JR Frayn KN. Fatty acids, obesity, and insulin resistance: time for a reevaluation. Diabetes (2011) 60(10):2441–9. 10.2337/db11-0425 21948998 PMC3178283

[17] Engfeldt P Arner P. Lipolysis in human adipocytes, effects of cell size, age and of regional differences. Horm Metab Res Suppl (1988) 19:26–9. 3069692

[18] Lionetti L Mollica MP Lombardi A Cavaliere G Gifuni G Barletta A. From chronic overnutrition to insulin resistance: the role of fat-storing capacity and inflammation. Nutr Metab Cardiovasc Dis (2009) 19(2):146–52. 10.1016/j.numecd.2008.10.010 19171470

[19] Eckel RH Grundy SM Zimmet PZ. The metabolic syndrome. Lancet (2005) 365(9468):1415–28. 10.1016/S0140-6736(05)66378-7 15836891

[20] Roden M Price TB Perseghin G Petersen KF Rothman DL Cline GW Mechanism of free fatty acid-induced insulin resistance in humans. J Clin Invest (1996) 97(12):2859–65. 10.1172/JCI118742 8675698 PMC507380

[21] McQuaid SE Hodson L Neville MJ Dennis AL Cheeseman J Humphreys SM Downregulation of adipose tissue fatty acid trafficking in obesity: a driver for ectopic fat deposition? Diabetes (2011) 60(1):47–55. 10.2337/db10-0867 20943748 PMC3012196

[22] Fielding BA Callow J Owen RM Samra JS Matthews DR Frayn KN. Postprandial lipemia: the origin of an early peak studied by specific dietary fatty acid intake during sequential meals. Am J Clin Nutr (1996) 63(1):36–41. 8604667 10.1093/ajcn/63.1.36

[23] Estruch R Ros E Salas-Salvado J Covas MI Corella D Aros F Primary prevention of cardiovascular disease with a Mediterranean diet. N Engl J Med (2013) 368(14):1279–90. 10.1056/NEJMoa1200303 23432189

[24] Salas-Salvadó J Bulló M Estruch R Ros E Covas MI Ibarrola-Jurado N Prevention of diabetes with Mediterranean diets: a subgroup analysis of a randomized trial. Ann Intern Med (2014) 160(1):1–10. 10.7326/m13-1725 24573661

[25] Hagenfeldt L Wahren J Pernow B Räf L. Uptake of individual free fatty acids by skeletal muscle and liver in man. J Clin Invest (1972) 51(9):2324–30. 10.1172/JCI107043 4639017 PMC292398

[26] Raclot T Langin D Lafontan M Groscolas R. Selective release of human adipocyte fatty acids according to molecular structure. Biochem J (1997) 324(Pt 3):911–5. 9210416 10.1042/bj3240911 PMC1218508

[27] Lennon R Pons D Sabin MA Wei C Shield JP Coward RJ Saturated fatty acids induce insulin resistance in human podocytes: implications for diabetic nephropathy. Nephrol Dial Transplant (2009) 24(11):3288–96. 10.1093/ndt/gfp302 19556298 PMC7614380

[28] Sieber J Lindenmeyer MT Kampe K Campbell KN Cohen CD Hopfer H Regulation of podocyte survival and endoplasmic reticulum stress by fatty acids. Am J Physiol Renal Physiol (2010) 299(4):F821–9. 10.1152/ajprenal.00196.2010 20668104 PMC2957252

[29] Prentki M Madiraju SR. Glycerolipid metabolism and signaling in health and disease. Endocr Rev (2008) 29(6):647–76. 10.1210/er.2008-0007 18606873

[30] Novgorodov SA Szulc ZM Luberto C Jones JA Bielawski J Bielawska A Positively charged ceramide is a potent inducer of mitochondrial permeabilization. J Biol Chem (2005) 280(16):16096–105. 10.1074/jbc.M411707200 15722351

[31] Galadari S Rahman A Pallichankandy S Galadari A Thayyullathil F. Role of ceramide in diabetes mellitus: evidence and mechanisms. Lipids Health Dis (2013) 12:98. 10.1186/1476-511X-12-98 23835113 PMC3716967

[32] Griner EM Kazanietz MG. Protein kinase C and other diacylglycerol effectors in cancer. Nat Rev Cancer (2007) 7(4):281–94. 10.1038/nrc2110 17384583

[33] Maedler K Spinas GA Dyntar D Moritz W Kaiser N Donath MY. Distinct effects of saturated and monounsaturated fatty acids on beta-cell turnover and function. Diabetes (2001) 50(1):69–76. 10.2337/diabetes.50.1.69 11147797

[34] Listenberger LL Ory DS Schaffer JE. Palmitate-induced apoptosis can occur through a ceramide-independent pathway. J Biol Chem (2001) 276(18):14890–5. 10.1074/jbc.M010286200 11278654

[35] Nolan CJ Larter CZ. Lipotoxicity: why do saturated fatty acids cause and monounsaturates protect against it? J Gastroenterol Hepatol (2009) 24(5):703–6. 10.1111/j.1440-1746.2009.05823.x 19646010

[36] Baldwin AC Green CD Olson LK Moxley MA Corbett JA. A role for aberrant protein palmitoylation in FFA-induced ER stress and β-cell death. Am J Physiol Endocrinol Metab (2012) 302(11):E1390–8. 10.1152/ajpendo.00519.2011 22436701 PMC3378068

[37] Sieber J Weins A Kampe K Gruber S Lindenmeyer MT Cohen CD Susceptibility of podocytes to palmitic acid is regulated by stearoyl-CoA desaturases 1 and 2. Am J Pathol (2013) 183(3):735–44. 10.1016/j.ajpath.2013.05.023 23867797 PMC3763774

[38] Kampe K Sieber J Orellana JM Mundel P Jehle AW. Susceptibility of podocytes to palmitic acid is regulated by fatty acid oxidation and inversely depends on acetyl-CoA carboxylases 1 and 2. Am J Physiol Renal Physiol (2014) 306(4):F401–9. 10.1152/ajprenal.00454.2013 24338821 PMC3920022

[39] Nakamura S Takamura T Matsuzawa-Nagata N Takayama H Misu H Noda H Palmitate induces insulin resistance in H4IIEC3 hepatocytes through reactive oxygen species produced by mitochondria. J Biol Chem (2009) 284(22):14809–18. 10.1074/jbc.M901488200 19332540 PMC2685662

[40] Solinas G Naugler W Galimi F Lee MS Karin M. Saturated fatty acids inhibit induction of insulin gene transcription by JNK-mediated phosphorylation of insulin-receptor substrates. Proc Natl Acad Sci U S A (2006) 103(44):16454–9. 10.1073/pnas.0607626103 17050683 PMC1637603

[41] Turban S Hajduch E. Protein kinase C isoforms: mediators of reactive lipid metabolites in the development of insulin resistance. FEBS Lett (2011) 585(2):269–74. 10.1016/j.febslet.2010.12.022 21176778

[42] Mima A Ohshiro Y Kitada M Matsumoto M Geraldes P Li C Glomerular-specific protein kinase C-β-induced insulin receptor substrate-1 dysfunction and insulin resistance in rat models of diabetes and obesity. Kidney Int (2011) 79(8):883–96. 10.1038/ki.2010.526 21228767 PMC3612886

[43] Welsh GI Hale LJ Eremina V Jeansson M Maezawa Y Lennon R Insulin signaling to the glomerular podocyte is critical for normal kidney function. Cell Metab (2010) 12(4):329–40. 10.1016/j.cmet.2010.08.015 20889126 PMC4949331

[44] Back SH Kaufman RJ. Endoplasmic reticulum stress and type 2 diabetes. Annu Rev Biochem (2012) 81:767–93. 10.1146/annurev-biochem-072909-095555 22443930 PMC3684428

[45] Ozcan U Yilmaz E Ozcan L Furuhashi M Vaillancourt E Smith RO Chemical chaperones reduce ER stress and restore glucose homeostasis in a mouse model of type 2 diabetes. Science (2006) 313(5790):1137–40. 10.1126/science.1128294 16931765 PMC4741373

[46] Lindenmeyer MT Rastaldi MP Ikehata M Neusser MA Kretzler M Cohen CD Proteinuria and hyperglycemia induce endoplasmic reticulum stress. J Am Soc Nephrol (2008) 19(11):2225–36. 10.1681/ASN.2007121313 18776125 PMC2573014

[47] Cunard R Sharma K. The endoplasmic reticulum stress response and diabetic kidney disease. Am J Physiol Renal Physiol (2011) 300(5):F1054–61. 10.1152/ajprenal.00021.2011 21345978 PMC3094049

[48] Luo ZF Feng B Mu J Qi W Zeng W Guo YH Effects of 4-phenylbutyric acid on the process and development of diabetic nephropathy induced in rats by streptozotocin: regulation of endoplasmic reticulum stress-oxidative activation. Toxicol Appl Pharmacol (2010) 246(1–2):49–57. 10.1016/j.taap.2010.04.005 20399799

[49] Qi W Mu J Luo ZF Zeng W Guo YH Pang Q Attenuation of diabetic nephropathy in diabetes rats induced by streptozotocin by regulating the endoplasmic reticulum stress inflammatory response. Metabolism (2011) 60(5):594–603. 10.1016/j.metabol.2010.07.021 20817186

[50] Kaufman RJ. Orchestrating the unfolded protein response in health and disease. J Clin Invest (2002) 110(10):1389–98. 10.1172/JCI200216886 12438434 PMC151822

[51] Ma Y Hendershot LM. The unfolding tale of the unfolded protein response. Cell (2001) 107(7):827–30. 10.1016/S0092-8674(01)00623-7 11779459

[52] Rasheva VI Domingos PM. Cellular responses to endoplasmic reticulum stress and apoptosis. Apoptosis (2009) 14(8):996–1007. 10.1007/s10495-009-0341-y 19360473

[53] Travers KJ Patil CK Wodicka L Lockhart DJ Weissman JS Walter P. Functional and genomic analyses reveal an essential coordination between the unfolded protein response and ER-associated degradation. Cell (2000) 101(3):249–58. 10.1016/S0092-8674(00)80835-1 10847680

[54] Lee AH Iwakoshi NN Glimcher LH. XBP-1 regulates a subset of endoplasmic reticulum resident chaperone genes in the unfolded protein response. Mol Cell Biol (2003) 23(21):7448–59. 10.1128/MCB.23.23.8913-8923.2003 14559994 PMC207643

[55] Acosta-Alvear D Zhou Y Blais A Tsikitis M Lents NH Arias C XBP1 controls diverse cell type- and condition-specific transcriptional regulatory networks. Mol Cell (2007) 27(1):53–66. 10.1016/j.molcel.2007.06.011 17612490

[56] Laybutt DR Preston AM Akerfeldt MC Kench JG Busch AK Biankin AV Endoplasmic reticulum stress contributes to beta cell apoptosis in type 2 diabetes. Diabetologia (2007) 50(4):752–63. 10.1007/s00125-007-0749-2 17268797

[57] Yasuda M Tanaka Y Kume S Morita Y Chin-Kanasaki M Araki H Fatty acids are novel nutrient factors to regulate mTORC1 lysosomal localization and apoptosis in podocytes. Biochim Biophys Acta (2014) 1842(7):1097–108. 10.1016/j.bbadis.2014.04.001 24726883

[58] Wu J Zhang R Torreggiani M Ting A Xiong H Striker GE Induction of diabetes in aged C57B6 mice results in severe nephropathy: an association with oxidative stress, endoplasmic reticulum stress, and inflammation. Am J Pathol (2010) 176(5):2163–76. 10.2353/ajpath.2010.090386 20363923 PMC2861082

[59] Oyadomari S Mori M. Roles of CHOP/GADD153 in endoplasmic reticulum stress. Cell Death Differ (2004) 11(4):381–9. 10.1038/sj.cdd.4401373 14685163

[60] Marciniak SJ Yun CY Oyadomari S Novoa I Zhang Y Jungreis R CHOP induces death by promoting protein synthesis and oxidation in the stressed endoplasmic reticulum. Genes Dev (2004) 18(24):3066–77. 10.1101/gad.1250704 15601821 PMC535917

[61] Yamaguchi H Wang HG. CHOP is involved in endoplasmic reticulum stress-induced apoptosis by enhancing DR5 expression in human carcinoma cells. J Biol Chem (2004) 279(44):45495–502. 10.1074/jbc.M406933200 15322075

[62] Li G Mongillo M Chin KT Harding H Ron D Marks AR Role of ERO1-alpha-mediated stimulation of inositol 1,4,5-triphosphate receptor activity in endoplasmic reticulum stress-induced apoptosis. J Cell Biol (2009) 186(6):783–92. 10.1083/jcb.200904060 19752026 PMC2753154

[63] McCullough KD Martindale JL Klotz LO Aw TY Holbrook NJ. Gadd153 sensitizes cells to endoplasmic reticulum stress by down-regulating Bcl2 and perturbing the cellular redox state. Mol Cell Biol (2001) 21(4):1249–59. 10.1128/MCB.21.4.1249-1259.2001 11158311 PMC99578

[64] Borradaile NM Han X Harp JD Gale SE Ory DS Schaffer JE. Disruption of endoplasmic reticulum structure and integrity in lipotoxic cell death. J Lipid Res (2006) 47(12):2726–37. 10.1194/jlr.M600299-JLR200 16960261

[65] Promlek T Ishiwata-Kimata Y Shido M Sakuramoto M Kohno K Kimata Y. Membrane aberrancy and unfolded proteins activate the endoplasmic reticulum stress sensor Ire1 in different ways. Mol Biol Cell (2011) 22(18):3520–32. 10.1091/mbc.E11-04-0295 21775630 PMC3172275

[66] Preston AM Gurisik E Bartley C Laybutt DR Biden TJ. Reduced endoplasmic reticulum (ER)-to-Golgi protein trafficking contributes to ER stress in lipotoxic mouse beta cells by promoting protein overload. Diabetologia (2009) 52(11):2369–73. 10.1007/s00125-009-1506-5 19727664

[67] Maurel M Chevet E Tavernier J Gerlo S. Getting RIDD of RNA: IRE1 in cell fate regulation. Trends Biochem Sci (2014) 39(5):245–54. 10.1016/j.tibs.2014.02.008 24657016

[68] Chen Y Brandizzi F. IRE1: ER stress sensor and cell fate executor. Trends Cell Biol (2013) 23(11):547–55. 10.1016/j.tcb.2013.06.005 23880584 PMC3818365

[69] Guo ZK Jensen MD. Accelerated intramyocellular triglyceride synthesis in skeletal muscle of high-fat-induced obese rats. Int J Obes Relat Metab Disord (2003) 27(9):1014–9. 10.1038/sj.ijo.0802380 12917705

[70] Zhang XJ Chinkes DL Wu Z Herndon DN Wolfe RR. The synthetic rate of muscle triglyceride but not phospholipid is increased in obese rabbits. Metabolism (2009) 58(11):1649–56. 10.1016/j.metabol.2009.05.021 19608209

[71] Maeda S Kobayashi MA Araki S Babazono T Freedman BI Bostrom MA A single nucleotide polymorphism within the acetyl-coenzyme A carboxylase beta gene is associated with proteinuria in patients with type 2 diabetes. PLoS Genet (2010) 6(2):e1000842. 10.1371/journal.pgen.1000842 20168990 PMC2820513

[72] Tang SC Leung VT Chan LY Wong SS Chu DW Leung JC The acetyl-coenzyme A carboxylase beta (ACACB) gene is associated with nephropathy in Chinese patients with type 2 diabetes. Nephrol Dial Transplant (2010) 25(12):3931–4. 10.1093/ndt/gfq303 20519229 PMC3108368

[73] Shah VN Cheema BS Sharma R Khullar M Kohli HS Ahluwalia TS ACACbeta gene (rs2268388) and AGTR1 gene (rs5186) polymorphism and the risk of nephropathy in Asian Indian patients with type 2 diabetes. Mol Cell Biochem (2013) 372(1–2):191–8. 10.1007/s11010-012-1460-2 23081748

[74] Sharma K Ramachandrarao S Qiu G Usui HK Zhu Y Dunn SR Adiponectin regulates albuminuria and podocyte function in mice. J Clin Invest (2008) 118(5):1645–56. 10.1172/JCI32691 18431508 PMC2323186

[75] Listenberger LL Han X Lewis SE Cases S Farese RV Ory DS Triglyceride accumulation protects against fatty acid-induced lipotoxicity. Proc Natl Acad Sci U S A (2003) 100(6):3077–82. 10.1073/pnas.0630588100 12629214 PMC152249

[76] Yen CL Stone SJ Koliwad S Harris C Farese RV Jr. Thematic review series: glycerolipids. DGAT enzymes and triacylglycerol biosynthesis. J Lipid Res (2008) 49(11):2283–301. 10.1194/jlr.R800018-JLR200 18757836 PMC3837458

[77] Mittendorfer B. Origins of metabolic complications in obesity: adipose tissue and free fatty acid trafficking. Curr Opin Clin Nutr Metab Care (2011) 14(6):535–41. 10.1097/MCO.0b013e32834ad8b6 21849896 PMC3711689

[78] Goodpaster BH He J Watkins S Kelley DE. Skeletal muscle lipid content and insulin resistance: evidence for a paradox in endurance-trained athletes. J Clin Endocrinol Metab (2001) 86(12):5755–61. 10.1210/jcem.86.12.8075 11739435

[79] Liu L Shi X Bharadwaj KG Ikeda S Yamashita H Yagyu H DGAT1 expression increases heart triglyceride content but ameliorates lipotoxicity. J Biol Chem (2009) 284(52):36312–23. 10.1074/jbc.M109.049817 19778901 PMC2794747

[80] Benhamed F Denechaud PD Lemoine M Robichon C Moldes M Bertrand-Michel J The lipogenic transcription factor ChREBP dissociates hepatic steatosis from insulin resistance in mice and humans. J Clin Invest (2012) 122(6):2176–94. 10.1172/JCI41636 22546860 PMC3366390

[81] Jiang T Wang XX Scherzer P Wilson P Tallman J Takahashi H Farnesoid X receptor modulates renal lipid metabolism, fibrosis, and diabetic nephropathy. Diabetes (2007) 56(10):2485–93. 10.2337/db06-1642 17660268

[82] Wang XX Jiang T Shen Y Adorini L Pruzanski M Gonzalez FJ The farnesoid X receptor modulates renal lipid metabolism and diet-induced renal inflammation, fibrosis, and proteinuria. Am J Physiol Renal Physiol (2009) 297(6):F1587–96. 10.1152/ajprenal.00404.2009 19776172 PMC2801344

